# Nuclear envelope mechanobiology: linking the nuclear structure and function

**DOI:** 10.1080/19491034.2021.1962610

**Published:** 2021-08-30

**Authors:** Matthew Goelzer, Julianna Goelzer, Matthew L. Ferguson, Corey P. Neu, Gunes Uzer

**Affiliations:** a Materials Science and Engineering, Boise State University, Boise, ID, US; b Biomolecular Science, Boise State University, Boise, ID, US; c Physics, Boise State University, Boise, ID, US; d Paul M. Rady Department of Mechanical Engineering, University of Colorado, Boulder, CO, US; e Mechanical and Biomedical Engineering, Boise State University, Boise, ID, US

**Keywords:** Nuclear envelope, nuclear mechanics, mechanobiology, chromatin, live imaging

## Abstract

The nucleus, central to cellular activity, relies on both direct mechanical input as well as its molecular transducers to sense external stimuli and respond by regulating intra-nuclear chromatin organization that determines cell function and fate. In mesenchymal stem cells of musculoskeletal tissues, changes in nuclear structures are emerging as a key modulator of their differentiation and proliferation programs. In this review we will first introduce the structural elements of the nucleoskeleton and discuss the current literature on how nuclear structure and signaling are altered in relation to environmental and tissue level mechanical cues. We will focus on state-of-the-art techniques to apply mechanical force and methods to measure nuclear mechanics in conjunction with DNA, RNA, and protein visualization in living cells. Ultimately, combining real-time nuclear deformations and chromatin dynamics can be a powerful tool to study mechanisms of how forces affect the dynamics of genome function.

## Introduction

Cells both sense and adapt to dynamic mechanical environments in tissues. Cellular mechanosensation is accomplished through a variety of structures and proteins that reside within the plasma membrane, the cytoskeleton, and the nucleus. Depending on the type of sensory element and the external stimuli, mechanical signals are either converted into biochemical signaling cascades or physically transmitted to the intra-cellular structures (Table 1). This conversion of extracellular deformations into intra-cellular information is called mechanotransduction. For example, application of extracellular mechanical signals such as substrate strain first activates focal adhesions, protein plaques smaller than 200 nm comprised of integrins, focal adhesion kinase (FAK), talin, paxilin, vinculin, and zyxin that enable direct connections between the extracellular matrix (ECM) and the cell [1]. In stem cells, strain application recruits signaling complexes to focal adhesions, essentially turning them into intracellular signaling relays for extracellular mechanical information [2]. Upon mechanical challenge, more structural elements, such as vinculin, paxilin, and talin, as well as signaling molecules, including FAK, Src, and Akt, are recruited into focal adhesions [3–7]. These signaling events in focal adhesions in turn activate adaptations of cell cytoskeleton where compressive forces on microtubules balance the contractile pulling forces generated by F-actin stress fibers. Numerous proteins maintain the structural adaptation of the F-actin cytoskeleton, including actin related protein (Arp) 2/3 complexes that maintain branching [8], formin homology 1 & 2 domain containing proteins that regulate the end-to-end actin formation [9]. Changes in the F-actin contractility and tension are largely regulated by Rho GTPases, such as RhoA, Ras, and CDC42A [10]. RhoA, for example, recruits myosin light chain kinase to F-actin fibers through its effector protein ROCK, which in turn activates the dimerized motor protein myosin II to generate tension by pulling F-actin bundles together [11]. Not only these changes in cytoskeletal contractions are directly transmitted to cell nuclei through nuclear envelope proteins such as Linker of Nucleoskeleton and Cytoskeleton (LINC) complex [12], restructuring events also result in activation of a number of signaling molecules, most notably, β-catenin, and YAP/TAZ. Following strain application for example, both β-catenin and YAP are activated (de-phosphorylated) in the cytoplasm [13,14]. Following their activation by mechanical force both β-catenin [15,16] and YAP/TAZ [17–19] enter cell nuclei through nuclear pores to act as co-transcriptional factors for regulating cell function. Mechanical information, whether directly through cytoskeletal networks or through intermediate molecular transducers, has to be transmitted through the nuclear envelope and into the nucleus to direct cell function and fate.

**Table 1.** Common *in vitro* mechanical force stimulation methods and their major studied outcomes

Mechanical force	Description	Major outcomes	Benefits	Drawbacks
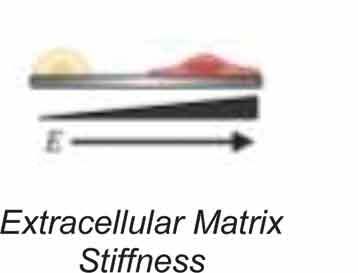	Stiffening or softening of extracellular matrix to induce mechanical responses similar to that of native tissue ^[124,134,240,241]^	• Focal adhesion activation • Actin cytoskeleton polymerization • Nuclear stiffening • Cell differentiation • Chromatin organization	• Replicates to native tissue mechanics • No additional apparatus required to induce mechanical signals • No additional apparatus required to induce mechanical signals	• Can have uneven stiffness profiles across surfaces • Harder to image live or fixed cells
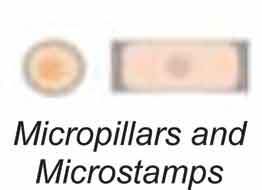	Restricting cell shape through physical impediments or shape of adherent surface ^[32–35,242]^	• Cytoskeleton & nucleus shape • Cell differentiation • Chromatin organization	• Easy to manufacture and implement • Isolates function of cell shape in cellular functions • Can image live or fixed cells	• Low cell density • Partial homology to tissue environment
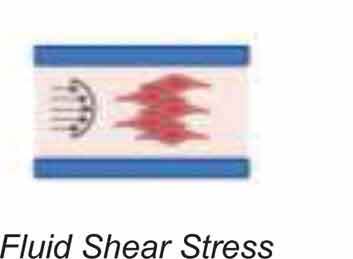	Mimicry of fluid shear stress forces found in vasculature systems ^[31,112–115,243,244]^	• Cell and nucleus orientation • Cytoskeleton remodeling	• High homology to vasculature forces • Easy to mimic human pathologies	• Requires use of specially designed bioreactors • Fluid force can be non- uniform between experiment sets
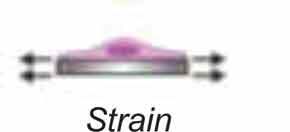 *Strain*	Stretching of adherent substrate to produce dynamic or static strain forces ^[6,7,13–17,37,52,56,100,127]^	• Actin cytoskeleton • Cell differentiation • Cell proliferation • Focal adhesion signaling • Nuclear signaling and structure • Chromatin organization	• Easy to use • Induces strong regulation of differentiation and stimulation of the actin cytoskeleton	• Requires expensive strain application machinery • Limited by size of specialized cell culture plates
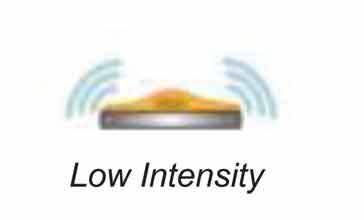	Low magnitude strain induced by low amplitude, high-frequency vibration ^[19,37,53,55,56,100]^	• Focal adhesions signaling • Cell differentiation • Cell proliferation • Nuclear signaling and structure	• Similar homology to muscle-induced vibration forces observed in native tissue • Can be utilized in cell culture, tissues, and mammalian models	• Requires custom-made bioreactors • Requires long-term exposure to mechanical signals • Less potent mechanical signal compared to strain and fluid shear
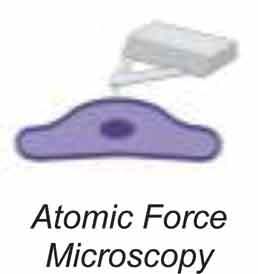	Probing of individual cells and nuclei with rounded-tip atomic force microscopy ^[100,145,147,169,245]^	• Measure Cell and nuclear stiffness • Force induced translocation of mechanically sensitive biomolecules	• Provides high resolution stiffness measurement of cells and nuclei • Targeted mechanical activation of mechanosensitive signaling pathways	• Require expensive equipment Challenging to provide provide population-based measurements • Hard to determine if measuring proper target versus non-desired targets
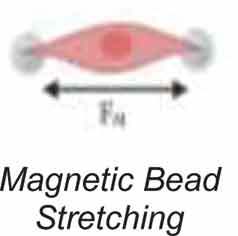	Use of magnetic beads to induce physical strain on individual cells ^[136,246–248]^	• Force induced translocation of mechanically sensitive biomolecules • Nuclei mechanoresponse •Actin cytoskeleton remodeling • Chromatin	• Allows for targeted strain on an individual cell level • Can induce targeted chromatin structure changes	• Does not provide population-based measurements • Requires use of special equipment

**Figure 1.**
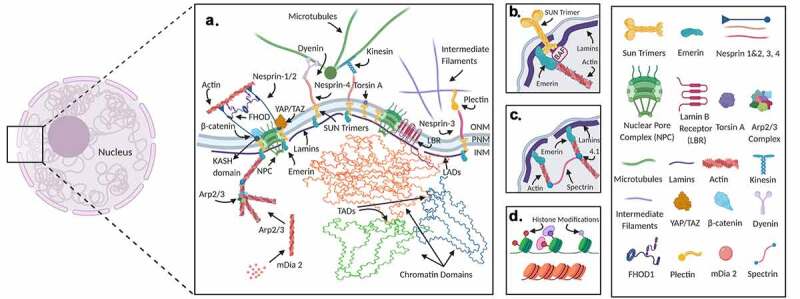
**Nucleus is a mechanically integrated mechanosignaling center**. Nuclear structural proteins interact with the cytoskeleton, chromatin, and the nuclear membrane to stabilize the nucleus and provide mechanosensing functions (Insert A). LINC complexes composed of Sun 1/2 trimers and Nesprin 1/2 mechanically couple the actin cytoskeleton. The LINC complex also interacts with nuclear pore complexes (NPC) and in-part regulate the access of important mechanical transducers such as β-catenin and YAP/TAZ into the nucleus. Nesprin-3 through interactions with plectin and nesprin-4 are also known to interact with cytoplasmic intermediate filaments and microtubules, respectively. Nesprins can also bind to microtubules via dynein and kinesin. Mechanical coupling of actin and the LINC complex involves cytoplasmic formins such as FHOD1 that attaches nesprins and actin at multiple points for a more robust association. Torsin A may also facilitate the LINC assembly at the nuclear envelope. A nuclear envelope transmembrane protein, Emerin connects the LINC complex, via SUN1/2 and nesprin-1/2 to the chromatin through BAF and lamin A/C (Insert B). Emerin also associates and plays a role in regulating extra and intranuclear actin. The intranuclear actin network is formed through the crosslinking of short F-actin fibers via protein 4.1 and spectrin that provides elastic structural properties to the nucleus (Insert C). Inside the nucleus, G-actin is assembled into linear and branched networks through regulatory proteins such as arp2/3 and mDia2 and influence chromatin dynamics and gene access. Chromatin domains that bind to the nuclear lamins are called lamin-associated-domains (LAD). These domains have been shown to be correlated with heterochromatin, producing repression of gene expression of genes in the LADs. These chromatin domains conserve epigenetic histone modifications. Changes of histone modifications, topologically associated domains (TADs), and LADs all result in changes in gene expression and cell differentiation (Insert D)

The nucleus, long thought to be just a simple and isolated house for the DNA of the cell, is now emerging as a far more intricate organelle with dynamic skeletal proteins and active subunits. This new view not only makes the nucleus a complex system but also a vital component that is integral to the overall cell function and genome regulation. Investigations into nuclear structure and function revealed that the nucleus has its own structural network called the nucleoskeleton, which for the purposes of this review will be defined as the insoluble fraction of the nuclei including nucleoskeletal proteins and chromatin but not RNA [20]. The nucleoskeleton component includes proteins such as the LINC complex, lamina proteins, emerin, and spectrins to name a few. The nucleoskeleton proteins are vital for the mechanical sensing of the cell and are the means by which the mechanical signal is transduced into the nucleus and ultimately to the chromatin regulating genome expression and chromosomal organization. While there have been great advances made in the last few decades, there is still much that is not understood about DNA, RNA, and protein dynamics in the nucleus. Here, we provide a review of recent literature of nuclear proteins implicated in mechanosignaling (Figure 1). The next two sections review the mechanical regulation of the nucleus by mechanical forces and highlight recent advances in quantifying real-time nuclear mechanics. Finally, we will introduce fluorescent labeling strategies that will make visualizing the DNA, RNA, and protein dynamics during mechanical stimulation possible, as well as cutting-edge microscopy techniques useful for quantifying biomolecular dynamics occurring in response to mechanical stimulation. Together, these technologies promise to provide invaluable information on the interplay between the nucleoskeleton proteins, gene expression, and functionality of the chromatin.

## Nuclear structure and mechanical force

### LINC complex

The Linker of Nucleoskeleton and Cytoskeleton (LINC) complex forms a physical link between the cytoskeleton and nucleus. Located in the nuclear envelope, the LINC complex is formed from multiple proteins that connect to actin, microtubules, and intermediate filaments in the cytoskeleton [21–24]. LINC complex proteins can be categorized into two main groups: those that are located on the outer nuclear membrane (ONM) forming connections to the cytoskeleton and span into the perinuclear space (PNS); and those that are located in the inner nuclear membrane creating connections between proteins inside the nucleus and LINC complex proteins in the ONM [21–24]. LINC complex proteins that form the first group are nesprin proteins. In mammalian cells, there are four main forms of nesprins, nesprins 1–4. While there are a number of smaller analogs of nesprins found elsewhere in the cell such as N-terminal nesprin-2 that binds to cell–cell junctions and actin [25], we will focus on the nesprins that facilitate nucleo-cytoskeletal connectivity and mechanosignaling. Nesprins bind to cytoskeletal elements via their N-termini protruding into the cytoplasm. Their C-termini extend into the PNS where a conserved KASH (Klarsicht, ANC-1, and Syne Homology) domain binds to other major LINC complex proteins called SUN proteins [21–24]. Other unique ONM proteins such as KASH5 and Jaw1 are involved in regulation of cell shape by binding to microtubules but their role in mechanosignaling requires further investigation [26,27]. Nesprins play an important role in mechanosignaling. During mechanical stimulation, the RhoA signaling pathway is activated, forming F-actin stress fibers over the nucleus creating an ‘actin cap’ [28–31]. Nesprins bind to these actin fibers and then regulate nuclear morphology, orientation, and motility [28–31]. Mechanical stimulation through regulation of cell shape increases the number of nesprin associations with the actin cap in both Human HUVAC [32–35] and mouse NIH-3T3 cells [32–35]. Depletion of nesprins negatively impacts mechanical response as actin cap does not form during shear stress [31] and mesenchymal stem cells (MSCs) are not able to mechanically activate osteogenesis through extracellular matrix (ECM) stiffening [36]. Furthermore, the loss of nesprins leads to the dysfunctional mechanoregulation of differentiation in MSCs, pushing their differentiation away from osteogenesis and into adipogenesis [36]. Interestingly, while substrate strain activates the focal adhesion signaling independent of nesprin function [6,37], strain-induced YAP nuclear entry is inhibited when nesprin-1 is depleted in stem cells [17]. These data indicate that nesprins provide a unique target that will allow for the investigation into nuclear mechanical signaling and mechanoresponse independent of cytoplasmic mechanoresponse events. While future research into the LINC complex via nesprins is needed, a considerable amount of research into the LINC complex SUN proteins has been done, which we will discuss next.

There are two main SUN proteins in the LINC complex in somatic mammalian cells, SUN1 and SUN2. The other SUN proteins SUN3-5 are also found in the LINC complex but are found mainly in germline cells [22,38,39]. SUN proteins are located in the INM and form trimers [40] that bind to the KASH domain of nesprins in the PNS via their C-terminal SUN domains, anchoring nesprins to the nuclear envelope [41,42]. Extending into the nucleus the N-terminal of SUN proteins binds to lamin A/C [41], emerin [43], and chromatin [44]. The LINC complex thus provides a physical connection between the cytoskeleton outside the nucleus and intranuclear actin and chromatin inside the nucleus via its interaction with emerin and barrier-to-autointegration factor (BAF) [23,45]. Depletion of SUN proteins disrupts centrosome orientation, nuclear positioning [46–48], and meiosis [36]. Important in these processes are microtubules. SUN proteins regulate microtubule-dependent DNA repair [49] and spindle formation [50]. Therefore, an important role of SUN proteins is the regulation of cell proliferation and meiosis. While one aspect of SUN protein effects is centered around microtubule regulation of proliferation, SUN proteins also regulate mechanical response. Mechanical stimulation via low-intensity vibration (LIV), strain, and ECM activates mechanically sensitive biomolecular pathways such as Yes-associated-protein (YAP) and β-catenin/Wnt pathways [6,13,18,51,52], that in turn regulate both proliferation and differentiation [18,37,51,53–57]. SUN proteins regulate mechanical response to strain and atomic force microscopy-induced cell deformation by restricting YAP [58] and β-catenin [16,59] entry into the nucleus by disrupting nuclear pore complex organization [60,61]. Additionally, SUN proteins are required for mechanoresponse and mechanoregulation of adipogensis in MSCs [37,53–56] during low-intensity vibration (LIV). Interestingly, de-coupling of nesprins and SUN proteins also inhibits mechanoresponse to LIV [37,53–56]. Decoupling of the LINC complex also decreases nuclear strain and deformation during microneedle manipulation indicating physical force transmission from the cytoskeleton into the nucleus is lost during loss of function of the LINC complex [48]. Additionally, isolated nuclei lose their ability to stiffen during magnetic bead displacement pulling on nesprin-1 during simultaneous SUN1 and SUN2 depletion [62]. However, strain can overcome the depletion of SUN proteins and decoupling of the LINC complex activating mechanosensitive pathways located at the focal adhesions and cytoskelton [37,48,53–56]. It is clear that the LINC complex is important for cellular functioning and mechanoreponsiveness, and is the lynchpin by which mechanical and biomolecular signals enter the nucleus. However, the LINC complex does not account for all regulatory mechanisms of mechanoreponse in the nucleus. Other factors such as chromatin and lamin A/C affect cellular outcomes due to mechanical signals. These other systems cannot be underestimated in their contribution to cellular mechanics and mechanoreponse and require further investigation in tandem with the LINC complex to determine their interconnected roles in mechanoresponse.

### Emerin

Emerin is a LEM-domain (LAP2β, emerin, MAN1) family protein that is found in the endoplasmic reticulum and in the nuclear envelope. In the nuclear envelope, emerin is found on the ONM and INM. Emerin is a pointed end actin capping protein that is capable of regulating actin dynamics in both intra and extra nuclear compartments [63]. SUN2 levels are significantly decreased in mutated emerin cells compared to wild type, playing a role in altered F-actin dynamics and nuclear structure [64]. Other emerin mutation isoforms cause mis-shaped nuclei, disorganized microtubule networks, and irregular cell shape [65]. Emerin’s role in mechanical signaling revolves around regulating nuclear stiffness and binding to the actin-cap. During nuclear tension via nesprin-1-coated magnetic tweezers, the tyrosine kinase Src is activated, which in turn Src phosphorylates emerin to increase nuclear stiffness. During emerin knockdown or expression of mutated, non-phosphorylated emerin, isolated nuclei do not experience nuclear stiffening during force application [62]. During mechanical strain, emerin increases its association with F-actin at the ONM and decreases its association with lamin A/C at the INM [66]. The mutated emerin isoform ∆K37 reduces actin-cap formation and actin organization in response to stiff substrates and cyclic strain [67]. While emerin regulates the physical connection of the nucleus to the cytoskeleton, its role has redundancy with that of the LINC complex. During LIV, depletion of emerin in MSCs does not impede mechanoactivation of the focal adhesions [37,53–56]. However, emerin has been shown to have a major impact on chromatin organization. As mentioned previously, emerin connects the LINC complex [43,68] to the chromatin through BAF and to lamin A [69]. As a result of this important connection, depleting emerin results in the dispersion of chromatin from the periphery to the center of the nucleus [70] potentially switching chromatin from facultative to constitutive states. Additionally, emerin-dependent switching of heterochromatin from H3K9me3 to H3K27me3 occurs during strain [66]. In DLD-1 cells, co-depletion of emerin and lamin A/C results in mislocalization of chromosomes [71]. Chromosome 19, which is positioned in the center of the nucleus, experiences relocalization to the periphery of the nucleus while chromosome 18 at the periphery sees no changes in positioning. Fluorescence recovery after photobleaching (FRAP) of H2A shows chromatin mobility increase of chromatin located internally of the nucleus which was aided by increased activity of nuclear myosin-1 (NM1) and nuclear actin during lamin A/C-emerin co-depletion [71]. The effects seen from the loss of emerin function range from loss of nuclear stiffness to chromatin organization, indicating emerin’s important role in the nuclear envelope. However, most of the effects from the loss of emerin also require other nuclear envelope and nucleoskeleton elements like that of lamin A/C and F-actin. This indicates that emerin’s involvement in regulating nuclear structure and mechanoreponse is more intricate than previously believed. Therefore, these interactions with chromatin, LINC complex, and lamin A/C must be further explored to fully understand emerin’s regulatory role in the nucleus during mechanical stimulation. Further insight into emerin’s potential role in regulating intra-nuclear actin should also be explored. As emerin associates with the actin-cap, regulates actin dynamics [63,64,72], and actin-driven nuclear positioning [73], emerin’s regulatory role on intranuclear actin could affect DNA repair and chromosome organization.

### Spectrin, intranuclear actin, and other nuclear proteins

Spectrins are tetramer proteins formed by association of two α–β heterodimers and are encoded in seven genes that are alternatively spliced to form different isoforms. Three types of spectrins are found in the nucleus: αII-spectrin, βIVΣ5-spectrin, and βII-spectrin, of which αII-spectrin is the most common [74]. Spectrin creates a network of nucleoskeleton proteins through crosslinking nuclear actin and protein 4.1, providing elastic properties as nuclei lacking αII-spectrin have decreased recovery of nuclei shape after compression [75]. Spectrin also plays an important role in DNA homologous recombination repair (HRR), nonhomologous end-joining (NHEJ), and nucleotide excision repair (NER) through recruiting DNA repair proteins to the repair site [76,77]. In addition to actin and protein 4.1, spectrins also associate with lamin A, lamin B, SUN2, emerin, and MYO1C. Knockdown of protein 4.1, a spectrin-actin stabilizer [78], results in nuclear blebbing and mislocalization of αII-spectrin, emerin, actin, and lamin A [74,79].

Actin is present in the nucleus as either monomeric G-actin or polymeric F-actin. The F-actin polymers in the nucleus differ from that of the cytoskeleton in that F-actin polymers in the nucleus form short, anti-parallel structures that are bound to lamin A, lamin B, and emerin [80]. Intra-nuclear actin binding to emerin causes intra-nuclear actin polymerization and is linked to localizing chromatin remodeling complexes [63,81]. Binding of F-actin to lamin A has also been associated with regulating actin polymerization as cells lacking lamin A form rod-like structures of F-actin in the nucleus [80]. G-actin monomers are required for proper DNA repair [82] and chromatin modifications [83,84]. While nuclei of Xenopus oocyte differs from mammalian nuclei, blocking intra-nuclear G-actin export out of the nucleus stabilizes nuclei and prevents nuclear rupture, indicative of increased mechanical competence [85]. Intra-nuclear F-actin also increases during cell spreading which is likely to exert complex loading on nuclei. Intranuclear F-actin formations due to cell spreading are prevented when lamin A/C, SUN1/2, or emerin are depleted [86]. Myosin motor proteins are also found in the nucleus and are unsurprisingly associated with the nuclear actin. Nuclear Myosin 1 (NM1) was the first nuclear myosin protein found in the nucleus and is an isoform of MYO1C produced by an alternative transcription start site of the *Myo1c* gene. Strain activates nuclear myosins and increases nuclear myosin localization to the INM, as well as increases of emerin-actin association. NM1 has been shown to be required for proper RNA polymerase I and II transcription through moving chromatin to transcription initiation sites [87–89]. When myosins I and V are depleted via RNAi, myosin I and V cannot relocalize to repair sites for heterochromatic double strand breaks [90]. While other myosin proteins have been found in the nucleus, their impact on nuclear function is still under investigation. Additionally, nuclear actin has a role in regulating chromatin organization and structure during mechanical stimulation, but this avenue of research has yet to be fully explored. Therefore, research into nuclear actin and other nuclear proteins should investigate their roles in regulating nuclear response to mechanical signals.

### Nuclear lamins

One family of nuclear proteins that has been extensively investigated are the lamins. The lamin family of proteins are type V intermediate filaments and consist of lamin A, lamin B, and lamin C. Alternative splicing of the *LMNA* gene produces either lamin A or lamin C [91] and together are termed A-type lamins. Another lamin family protein is lamin B which has three isoforms: lamin B1 encoded by *LMNB1* gene, lamin B2 and lamin B3 which are encoded by *LMNB2* and are formed via alternative splicing [92]. B-type lamins are found in all cell types, though lamin B3 is only found in spermatic cells [93–95]. Together, lamin A/C and lamin B proteins form the majority of the nuclear lamina located at the INM. Lamin A/C proteins associate with emerin, the LINC complex via SUN1/2, intranuclear actin, BAF, histones, and DNA [92,96]. Lamin B binds to emerin [97], intranuclear actin [80], DNA which is done through the nuclear envelope protein lamin binding receptor (LBR) [96,98], and other nuclear proteins [99]. Each lamin family protein has a distinct role in nuclear structure and function. During the loss of lamin A/C, the nucleus experiences blebbing, wrinkling, loss of circularity, increased volume, height, area, and decreased cellular and nuclei stiffening [100–104]. This loss of structural properties causes increased migration and proliferation [105–107]. Investigation into lamin A/C shows that during lamin A/C depletion fibroblasts are unable to harness apical F-actin fibers that are formed during substrate strain [30]. This inability to associate with F-actin fibers is also observed in progeria models. In progeria, a devastating early aging disease, a silent mutation in *LMNA* causes permanent farnesylation, preventing proteolytic cleavage causing progerin, a misfolded form of lamin A, to build up at the nuclear periphery [108,109]. *LMNA* mutation results in the increased phosphorylation of ERK1/2. *LMNA*-dependent phosphorylation of ERK1/2 causes the phosphorylation of FHOD1/3, inhibiting actin bundling at the nuclear envelope [110]. The regulatory role of lamin A/C in connecting to F-actin fibers results in the loss of nuclear positioning [110], nuclear movement [110], and negates jasplakinolide-induced nuclear F-actin formation in fibroblasts leading to reduced transcription [111]. These observations of lamin A/C loss and nuclear morphology alterations are constant throughout mechanical force stimulation. Fluid shear stress (FSS) is a common *in vitro* mechanical stimulation model to simulate both blood and interstitial fluid flow in tissues. Application of FSS *in vitro* causes remodeling of F-actin cytoskeleton [112–116]. *LMNA* -/- mouse embryonic fibroblasts (MEF) cells fail to form actin-cap associated F-actin fibers [31], suggesting an active role of LaminA/C in recruiting F-actin to nuclear surface in response to fluid shear [30]. Further corroborating with the idea that Lamin A/C may play a role in stabilizing nuclear envelope in response to mechanical force, when cells are elongated via rectangular microstamps, depletion of lamin A/C causes increased nuclei fluctuations when compared to control cells [34].

Unlike lamin A/C that is largely expressed in committed or multipotential cell types, lamin B is found in the brain cells of mice at birth and are expressed in early stages of embryonic development [98,117,118]. Similar to lamin-A/C-related laminopathies, while *LMNB1* and *LMNB2* are also linked to disease, very few if any diseases have been linked to mutations in the LMNB1 and LMNB2 genes. The best characterized disease is associated with the adult-onset leukodystrophy which causes demyelination of the central nervous system and is linked to duplication of *LMNB1*. Heterozygous mutation of *LMNB2* is linked to acquired partial lipodystrophy which presents as a loss of subcutaneous tissue in the neck, arms, legs, and face [119]. Depletion of lamin B results in chromatin instability and increased DNA double strand breaks [120], chromatin reorganization [121], and increased senescence similar to that of progeria [121]. Alterations to nuclear structure occur as well as increasing micronuclei [120] and nuclear rupture [122,123]. Lamin B has a critical role for the proper development of mice as *LMNB1* -/- mice experience die at birth and increased bone ossification [122]. Lamin B therefore has an important role in maintaining normal nuclear functioning. However, the role of lamin B during mechanical signaling is not as vital and is different from the role of lamin A/C. The role differences between lamin A/C and lamin B are largely seen during mechanical stimulation of the nucleus. Modulation of extracellular matrix (ECM) stiffness causes mechanical force effects on lamin A/C protein levels, lamin A/C structure, and nuclear lamina organization. Decreasing ECM stiffness decreases lamin A/C levels and causes re-localization of lamin A/C and lamin B into the interior of the nucleus [70] and causes the deformation and folding of lamin A/C [124,125]. In MSCs, ECM stiffness alters LBR:lamin A/C ratios. Softer extracellular matrices induce LBRs to be highly expressed relative to lamin A/C [126] correlating with increased adipogenesis while stiffer ECM induces a lower LBR/lamin A relationship pushing the MSCs to osteogenesis [126]. While these results show a role for lamin A/C, lamin B, and LBRs in mechanosensing pathways, cells with defective lamin B experience little changes in gene expression during mechanical stimulation [127] which further supports that lamin A/C is the main target to regulate mechanical signals and mechanoregulation. Indeed, further research into lamin A/C through microstamp cell shape regulation shows that cells forced into rectangular shapes increase lamin A association at the nuclear envelope [32], decrease nuclear size fluctuations [34], and induce osteogenic differentiation [128]. Contrastingly, cells forced into circular shapes have decreased lamin A association with nuclear envelope [32], large nucleus size fluctuations [34], increased chromatin and telomere diffusion [34], and inducement into adipogenesis [128]. Lamin A/C therefore has a more important role in regulating cellular and nuclear response to mechanical signals. However, we have shown that mechanoregulation of adipogenic differentiation in MSCs is independent of lamin A/C indicating that lamin A/C may have a limited or at least overlapping functionality with other nuclear proteins during mechanically induced repression of adipogenesis [100]. Further research into the role of the nuclear lamina, specifically, lamin A/C, is needed during mechanoregulation of differentiation in combination with other nuclear envelope elements such as emerin or the LINC complex to fully elucidate the full mechanoregulatory effects of nuclear envelope proteins.

### Chromatin

As the organized and packaged structure of histones and DNA, chromatin provides the nucleus with a mechanism to regulate not only genomic expression but also genomic organization and nuclear structural properties. Chromatin is known to associate with SUN proteins [44], emerin, lamin A/C through DNA binding domains and BAF, to lamin B via LBRs, and other nuclear proteins. Chromatin domains that are in proximity to and associated with the nuclear lamins are called lamin-associated-domains (LAD) [129,130] (Figure 1a). These domains have been shown to be correlated with heterochromatin, producing repression of gene expression of genes located in the LADs [131]. However, this model of LAD-mediated repression at the nuclear periphery does not account for the changes in the 3D chromatin organization observed under lamin depleted cells. Disabling the interaction of chromatin and nuclear lamins results in the loss of the inter- and intra-interactions between topological-associated domains (TADs) at both the periphery and internal regions of the nucleus [132]. Additionally, loss of lamin A/C alters chromatin diffusion [133]. Therefore, disabling the interaction of chromatin with the nuclear lamins not only affects the nuclear periphery but alters 3D organization of chromatin. Mechanical forces also regulate chromatin structure. Soft ECM induces increases in euchromatin [134] and localization of chromosomes 1, 18, and 19 to the nuclear interior, and upon replating on stiffer substrates only chromosome 18 experiences recovered localization [70]. Substrate strain causes an increase of heterochromatin and switching of heterochromatin from H3K9me3 to H3K27me3^66^[135]. Direct magnetic bead shear stress on the nucleus of Chinese hamster ovary (CHO) cells also shows that chromatin is induced into an open state and increases gene expression [136]. Depletion of SUN1/2, lamin B, lamin A/C, emerin, and BAF all cause similar chromatin movement and gene expression as magnetic bead shear stress [136]. Ultimately, these alterations of chromatin structure have major regulatory effects on differentiating stem cells. In MSCs, the heterochromatin marker H3K27me3 is decreased in cells differentiating into adipocytes, while the euchromatin markers H3K9ac, H3K4me3, and H4K5ac see an increase [51,137]. Alterations to chromatin are one of the first steps in cellular responses to mechanical signals. Understanding how stem cells alter their chromatin structure and organization in response to mechanical forces is required to truly understand and manipulate stem cell fate.

As the main house for DNA, it is a logical conclusion that both alteration to nuclei structure and mechanical force stimulation would alter chromatin. However, chromatin also has an important role in regulating the nuclear response to mechanical forces and regulating nuclear morphology. Disruption of chromatin structure via chromatin digestive MNase protein retards cell stiffening in response to low levels of strain displacement (<3 µm) [138]. Additionally, increases in heterochromatin induce nuclear stiffening [138,139] while increases in euchromatin result in decreased stiffness [138,139]. Reduced H1, a histone protein that stabilizes formation of condensed chromatin, does not alter heterochromatin markers but does result in decreased nuclear rigidity inducing increased nuclei fragility [140]. Additionally, decreased levels of heterochromatin also result in blebbing and protrusion of the nuclear envelope independent of lamin A/C [139–141]. Therefore, chromatin is a vital nuclear element that regulates gene expression, nuclear morphology, and nuclear mechanics. In order to fully understand how the nucleus responds to and senses mechanical signals, the interaction of chromatin and nuclear proteins must be further explored. Specifically, understanding the connections between chromatin and the nuclear envelope proteins is of great importance. As mechanical signals enter the nucleus through the nuclear envelope proteins, like that of the LINC complex, and are transferred to the chromatin, understanding the chromatin dynamics is of vital importance. A potential tool to investigate these dynamics is fluorescence microscopy, as the advancement of fluorescence microscopy beyond the diffraction limited spot has now provided a way to visualize these dynamics at the single molecule level, providing a launching point for further exploration and quantification of these changes that have not been achievable before.

## Characterization of nuclear structure and mechanics

The nucleus is a mechanosensitive organelle of the cell that allows for gene regulation and adaptation as an active response to biophysical stimuli from the cytoskeleton and surrounding environment. Numerous methodologies have been developed to probe nuclear structure and mechanics, including fluorescence anisotropy [142–144], micropipette aspiration [145,146], nanoindentation [147,148], and image-based assessment of aspect ratios [149,150], volume [151,152], deformable image registration [153,154], and deformation microscopy [155]. Characterization of bulk or local structure and mechanics is possible for isolated cells or nuclei, and additionally of cells embedded in two- and three-dimensional microenvironments. Like most biological structures, the nucleus is well-known to exhibit complex (e.g., nonlinear, time-dependent) properties, and available methods allow for the characterization of this behavior following a wide range of mechanical perturbations [62,156].

### Nuclear structure

Recent research reveals that the nuclear structure, with distinct euchromatin and heterochromatin subdomains, demonstrates a scale-dependent and solid-like behavior under some conditions that provides insight for the physical organization and regulation of the genome [157]. While microscopy methods like fluorescence microscopy and fluorescence recovery after photobleaching provide the ability to visualize the nuclear interior, additional methods are required to provide value-added characterization of nuclear structure. The morphology of the nucleus is commonly assessed based on measurement of the aspect ratio, volume, or a characteristic dimension such as major/minor axes [150,153,158]. Morphological analysis of this type commonly considers geometric changes of the nuclear periphery using automated or semi-automated algorithms and does not provide any intranuclear spatial information. A major strength of nuclear morphology measurements is the ability to assess large numbers of cells in a high-throughput manner, enabling population-level analysis of treatment responses, often at the cost of detailed intranuclear spatial information.

### Intranuclear strain

Local mechanical deformations, i.e., displacements and strains within the nuclear interior, may be related directly to altered transcriptional activities, possibly through the alteration and regulation of chromatin domains [159]. While the measurement of local deformation may reveal fundamental mechanobiological mechanisms, direct imaging of intranuclear mechanics is challenging. Commonly, fluorescent microscopy of viable cells is required to capture and tag the deforming nucleus in multiple (e.g., resting and mechanically loaded or stretched) states to allow for a description of motion of the nucleus in a ‘current’ configuration with respect to an initial ‘reference’ configuration. Widefield and confocal microscopy can be used to visualize living cells before and after deformation [154], and a natural extension of imaging modalities to include modern methods like super-resolution microscopy is possible.

Spatial mapping of deformation within the nucleus is accomplished using fluorescence anisotropy [160] and texture correlation [153,161]. Recently, deformation microscopy, based on hyperelastic warping and deformable image registration [155], demonstrated the ability to map biophysical and biochemical interactions due to substrate stiffness or hyperosmotic changes, or LINC disruption treatments, and have been used broadly to describe the mechanics of nuclei in cardiomyocytes, chondrocytes, and skeletal muscle *in vivo* [155,161,162]. Additionally, detailed strain patterns have been associated with distinct epigenetic modifications that impact development [163]. The use of hyperelasticity enables the measurement of complex nuclear behavior, including nonlinear elasticity in two and three dimensions, that would be expected to sufficiently describe intranuclear deformation for most anticipated applications. Certainly, nuclei have demonstrated extreme deformations, such as in migratory cancer cells in constrained geometries [164], and yet recovery of the nucleus is observed, aligning more with hyperelastic, and not plastic or permanent, deformation behavior.

### Intranuclear stiffness

Emerging methods also enable the description of the mechanical properties of heterochromatin and euchromatin domains. One method is intranuclear rheology [165,166] which tracks the passive movement of fiduciary markers such as fluorescent beads but may suffer from limitations including the possible invasive nature of bead insertion and the impact of embedded beads on cell viability. Recently, confocal Brillouin microscopy, a non-contact, direct readout of the viscoelastic properties of a material [167], has been applied to migrating tumor cells, which allows a real-time live cell metric for measuring stiffness changes in cell nuclei [168]. Atomic force microscopy with a needle-tip probe has recently demonstrated the ability to directly map the nuclear envelope and cell membrane stiffness within native tissue [169], and showed that the nuclear stiffness decreases with disruption of the extracellular matrix in living tissues, further emphasizing the physical links connecting the nucleus to the surrounding microenvironment. Optical microscopy-based [170–172] elastography is a powerful potential method to measure the distribution of mechanical properties noninvasively within the nucleus. Based on techniques like deformable image registration and inverse finite element methods, image-based elastography of heterochromatin and euchromatin domains in the deforming cell nucleus is now possible [173,174].

### Linking nuclear mechanics and mechanobiology

While characterization of the nucleus structure and mechanics is possible using numerous methods, still lacking are studies that carefully link biomechanics with cell and nuclear biological activity. Methods are required that allow for the rapid acquisition of biomechanical data coupled simultaneously with techniques that capture activities like rapid gene expression in response to mechanical loading. High spatial resolution imaging is needed to probe the single-cell level, ideally in complex three-dimensional microenvironments like hydrogels or native tissue. New methods explore combinatorial methods, including the use of photobleaching with unique Förster Resonance Energy Transfer (FRET) pairs [175,176], or deformable image registration with independent assessments of histone modifications or LINC disruption [163].

## Visualizing chromatin dynamics in living cells

In the sections leading here, we have detailed the mechano-responsive structures that make up nucleus as well as methods to apply mechanical force as well as methods to measure nuclear mechanics. While it is accepted that 3D structure and function of the nucleus and chromatin are inherently connected, ‘seeing is believing’[177], and therefore visualizing is critical to understand the structure and function of the genome. There are an increasing number of studies aimed at understanding how mechanical signals regulate nuclear mechanics at higher resolution, while at the same time there are several state-of-the-art optical techniques under-utilized in the field of mechanobiology that are capable of visualizing nuclear dynamics. In this section, we will first discuss possible approaches that can be combined to perform correlative measurements of mechanical stimulation and gene expression at high resolution as these may provide critical information about the relationship between mechanics and spatiotemporal (3D+1D) dynamics of the nucleus. Finally, we will focus on current methods of labeling DNA, RNA, and proteins in living cells and discuss details of different imaging modalities that can be used to discern the motion of these labeled structures.

### Fluorescence imaging techniques

For the study of living cells and tissues there is no substitute for light microscopy. The limited interaction of photons with biological matter combined with superb contrast provided by fluorescent labeling allows us to study both the prevalence and subcellular organization of selected biomolecules within living cells and tissues. The ever-growing list of highly specific fluorescent labels makes fluorescence microscopy one of the techniques of choice for studying nuclear architecture and function [178]. In the last decade the nucleus, which was a proverbial black box, has been unmasked as a highly dynamic, ultra-structured entity that is dynamically reforming based on biochemical cues from the microenvironment and mechanical cues from the tissue. This evolution of scientific understanding is in large part due to advances in light microscopy and new creative imaging techniques **[**179,180**]**.

The methods we will discuss here can provide information about nuclear structure and mechanics. One of the main methods is visualizing tracer particles. Depending upon its size, a tracer particle may sample and provide information on either the micro or macro environment of the local nuclear region through the generalized Stokes-Einstein equation [181]. Confinement of a particle within a region of the nucleus may also allow determination of phase separated domains which have been reported to correlate with specific histone modifications and transcriptional activity [182,183]. Methods such as fluorescence anisotropy can also characterize properties of the local environment of a tracer particle. If mechanical stimulus is applied to the nucleus, particle image velocimetry can be used as a control to quantify the applied stress or strain rate. Microrheology may be applied after mechanical stimulus to determine its effect on the local nuclear environment of a tracer particle [184]. Another more novel application in fluorescence microscopy is to monitor changes in gene expression affected by mechanical stimulus. It may be that in some cases there is a direct relationship between gene activation or repression and the mechanical environment of the nucleus. While this effect is well known in population measurements of stem cell differentiation [185], it has never been directly verified at the single cell or single molecule level.

As with determining the appropriate fluorescent label for the experimental question, there are a variety of labeling techniques with benefits and drawbacks. Some focus on temporal resolution at the expense of spatial resolution. Others are focused on determining molecular interactions and binding events. The below chart provides an overview of techniques that are available and useful in determining the structure and function of nuclear architecture and its role in nuclei’s mechanoresponsonse (Table 2). We will then further highlight several methods that promise to be valuable.

**Table 2.** Fluorescence imaging techniques

Technique	Description	Benefits	Drawbacks
*Colocalization*	The observation of spatial overlap between different fluorescent labels, which reveals associations and interactions between two molecules ^[249,250]^	• Can be conducted on widefield, confocal, and superresolution microscopes • Shows biomolecular associations and co-distributions	• Limited spatial and temporal resolution • Limited by resolution as the colocalization of two probes does not always signify association.
*Fluorescence Recovery After Photobleaching (FRAP)*	FRAP is used to determine the kinetics and diffusion of various biomolecules by intentionally photobleaching a portion of the sample and then observing how the fluorescence distribution returns to its previous state ^[71,251–254]^	• Useful for finding ratios of bound and unbound molecules, as well as protein mobility • Turns photobleaching, which is generally avoided, into a desirable	• The photobleaching process can be destructive to the sample because of the high light intensity • Sometimes incomplete fluorescence recovery occurs due to obstruction of diffusion • A local temperature increase at the photobleached site can affect the calculated diffusion rate [^255]^
*Fluorescence Correlation Spectroscopy (FCS)*	FCS utilizes fluctuations in fluorescence intensity in small detection volumes in samples of low concentration to investigate molecular dynamics ^[186–194]^	• Kinetics data can be measured in a living cell • Number of molecules of interest and their molecular brightness can be calculated	• Requires high labeling efficiency in order to get accurate kinetics data • Only counts the molecules in the observation volume, not the entire field of view
*Single Particle Tracking (SPT)*	SPT is a microscopy tool that allows the movement of individual particles to be followed within living cells. It provides information on molecular dynamics over time ^[256,257]^	• Monitors the trajectories of individual biomolecules in living cells • Good for studying localization dynamics	• Requires extremely low fluorescent background and very bright labels • Requires highly sensitive cameras • Requires TIRF or HILO microscopes • Photobleaching (due to widefield imaging)
*3D Orbital Tracking*	3D Orbital Tracking uses an unique scanning pattern. Instead of exciting the molecule directly, the laser passing around the bright spot indirectly excites it, resulting in a longer imaging window ^[187,214]^	• Minimal photobleaching • Can collect data for long periods of time	• Can only track one particle at a time • Only collects data on the molecule being tracked, not the rest of the field of view
*Förster Resonance Energy Transfer (FRET)*	FRET exploits the energy transfer that occurs between two chromophores that are in close proximity. The donor when in an excited state can transfer its energy to the acceptor through dipole-dipole coupling [^258]^. The excitation is accompanied by light emission and the transfer of energy is characterized by a loss of light emission. The efficiency of this transfer can be used to calculate small changes in distance between the chromophores [^259]^.	• FRET is a nondestructive spectroscopic technique • Characterized molecular interactions with high accuracy (on the 1–10 nm scale)	• Low signal-to-noise ratio • Sensitivity of probes to pH, temperature, ionic concentration, etc.
*Fluorescence Lifetime Imaging (FLIM)*	FLIM specifically measures how long a fluorophore stays in an excited state before emitting a photon ^[260,261]^	• Can detect molecular variations of fluorophores that are not apparent with spectral techniques alone • Ideal tool for removing background fluorescence intensity • Collects lifetime measurements for every pixel within the image	• Difficult to conduct in live cells because there are not enough photos per pixel • Requires in-depth data analysis

Fluorescence Correlation Spectroscopy (FCS) utilizes fluctuations in fluorescence intensity in small detection volumes in samples of low concentration to investigate molecular dynamics namely, diffusion, molecular conformations, binding events, and chemical reaction kinetics [186]. It was first developed by Elliot, Magde, and Webb [186] and later developed by Gratton et al. [187–189], Schwille et al. [190–194] and many others for scanning multiple labels and two photon excitation and was eventually extended to the study of transcription [195], translation [196], and splicing [197], and more recently gene activation [198,199]. FCS is conducted by measuring fluctuations in fluorescence intensity as fluorescent molecules enter and exit an illuminated space. Large jumps in intensity signify larger molecules or multiplexes as opposed to small jumps in intensity that signify smaller, individual molecules. Similarly, slow changes in intensity indicate slower moving, often larger molecules, while quick fluctuations in intensity indicate faster moving, often smaller molecules. FCS calculations are done using a correlation curve from the fluctuations in intensity. The taller the curve, the lower the concentration of molecules within the observation volume. The longer the curve, the slower they are moving [186]. FCS was originally conducted on homogenized samples in a cuvette; now this technique has been extended for use in live cell microscopy [200]. The cell now acts as the confined space like the cuvette. Not only can single biomolecules be analyzed through FCS, but multiple molecules can be studied simultaneously, and their intermolecular interactions can be quantified as well by using fluorescence cross correlation spectroscopy (FCCS) [189,201]. FCCS has been used extensively to quantify the kinetics of transcription factor binding and elongation as well as many other biomolecular interactions within the nucleus [202–204].

Single Particle Tracking (SPT) is a method that requires bright and stable fluorescent labeling, highly sensitive CCD or sCMOS cameras, and extremely low fluorescent background. In living cells this can only be achieved using a Total Internal Reflection Fluorescence (TIRF) [205,206] or Highly inclined illuminated optical sheet (HILO) [207] microscopes. SPT can be useful in determining the trajectories of individual particles with nanometer precision providing dynamic information about biomolecule locations. One of the major challenges with SPT is photobleaching. Even with improved fluorophores photobleaching often occurs within seconds or at most minutes on a widefield microscope, reducing the temporal resolution of correlative measurements. Recent advances have been made in this area with the development of lattice light sheet [208] and other microscopy methods [209–212], and has also been addressed by combining SPT with FCS and 3D Orbital Tracking [195,197–199]. This synergistic approach has been successfully used to visualize transcription factor binding dynamics [213].

3D Orbital Tracking, which was developed in 2005 by Levi and Gratton et al. [214,215], gets around photobleaching issues by changing the laser scanning pattern from x-y to a circular orbit [214]. Instead of exciting the molecule directly, the laser passing around the bright spot indirectly excites it, resulting in a longer imaging window [187,214]. This method has been used to acquire quantitative, single-cell, live data on transcription factor binding and elongation [198,199], as well as study lysosome active transport and free diffusion [214,216]. In addition to information on transcription factor binding and transcriptional activity, a laplace transformation of the mean squared displacement (MSD) of the 3D trajectory of a gene locus by orbital tracking may also give information on the complex viscoelastic modulus of the nuclear compartment [217].

Moving forward it is becoming increasingly necessary to combine these techniques to both validate findings as well as discover new information about nuclear structure and dynamics. By combining techniques, both spatially and temporally relevant data can be gleaned. FRAP and FRET are being used in conjunction to determine the dynamics of BAF and emerin interactions [218]. Colocalization and FRAP together showed that the crosstalk seen between the cytoskeleton and the nucleus is in large part regulated by lamin A/C and emerin modulating structural cytoskeletal proteins like actin [71]. FCCS and 3D Orbital tracking have been used synergistically to determine the kinetics of transcription factor binding and RNA synthesis [198]. It is not enough to solely study RNA, DNA–Protein interactions, or chromatin–chromatin interactions; each must be combined to understand how nuclear structure and gene expression are affected by mechanical and environmental cues. Not only is it powerful to combine two imaging techniques or two sequencing techniques, when both sequencing and imaging are combined unique research questions can be addressed.

### Fluorescent biomolecule labeling

There are a variety of labeling strategies available for visualizing biomolecules. Each provides varying pros and cons, making them ideal for different experimental questions. Some questions to consider when choosing a label method include: Is the experimental imaging going to be performed in live cells? How bright does my fluorophore need to be? Do I want the flexibility of adding my probe before each experiment or do I want the stability of having a self-labeling cell line? How important is fluorescent background and labeling efficiency? Based on the answers to these questions, the proper labeling method for your experiment can be identified. While well-established methods such as LacR [219] and MS2/PP7 [220] are powerful, readers are referred to Table 3 for an extensive list of methods that are available to researchers. Below, we highlight the most promising methods for imaging the nucleus while it undergoes mechanical stimulation.

**Table 3.** Fluorescence labeling technologies and their benefits and drawbacks

Label	Target biomolecule	Description	Benefits	Drawbacks
*DNA Binding Dyes (DAPI, Hoechst, SiR-DNA, and SPY650)*	DNA	These dyes fluoresce when they intercalate into the minor groove of DNA ^[262–264]^	• Requires minimal sample preparation • Labels all DNA indiscriminately	• Cannot label specific genes
*FiSH*	DNA/RNA	Fluorescence in-situ hybridization (FiSH) labels gene loci or RNA specifically with fluorescently labeled single stranded probes ^[265,266]^	• Labels DNA gene loci or RNA specifically • Multiple gene loci labeled at one time	• Cannot be used for live cell imaging • Requires specific probe design
*LacR & TetR*	DNA	LacR and TetR specifically label chromatin locus in living cells with a GFP-fusion protein ^[267,268]^	• Results in stable cell line that can be used over and over • Specific gene loci and individual gene loci can be imaged in live cells over multiple generation without the addition of probes	• Requires integration of prokaryotic operon sequences into the DNA • The gene editing may result in abnormal gene expression profiles
*dCas9*	DNA	dCas9 uses the CRISPR gene editing system for DNA labeling with a fluorescently tagged nuclease dead Cas9 in combination with specifically engineered guide RNAs ^[221–228]^.	• Live cell imaging without laborious or disruptive gene editing • Multiple gene loci labeled at one time • Ideal for studying chromatin dynamics	• Requires multiple CRISPR/Cas9 to produce a bright enough signal for imaging • The binding affinity of CRISPR/Cas9 is highly dependent upon the gRNA sequence
*MS2/PP7*	RNA	Fluorescent molecules bind to repetitive stem loops that have been introduced into the gene of interest. Each stem loop, of which there are often up to 24 copies, binds to a dimer of a chimeric protein composed of the phage protein, a nuclear localization signal and a fluorescent protein [197,269].	• Actively transcribing RNA can be imaged in real-time within a cell • Since MS2-RNA and PP7-RNA are sequence specific, both can be used simultaneously within a given cell, allowing for multiple RNAs to be visualized at the same time.	• Can only be used to label two distinct RNAs at a time • The multimerization of the stem loops results in a bulky label that can alter RNA kinetics
*dCas13*	RNA	dCas13 uses the CRISPR gene editing system for RNA labeling with a nuclease dead Cas13 in combination with specifically engineered guide RNAs ^[222,229]^ . Either the gRNA or the Cas13 can be fluorescently tagged.	• Versatile method for labeling RNA’s which have not been modified through the insertion of an RNA hairpin or other sequence • Sequence specific • Ideal for studying RNA dynamics	• Requires multiple copies of the RNA of interest and multiple CRISPR/Cas13 to produce a bright enough signal for imaging • The binding affinity of CRISPR/Cas13 is highly dependent upon the gRNA sequence
*RNA Aptamers*	RNA	RNA aptamers, like RNA Mango [^230]^, are sequences designed as molecular beacons and selected through SELEX ^[233,234]^. The resulting aptamer is capable of binding specific fluorophore derivatives with nanomolar affinity.	• Provides a fluorescence enhancement upon binding (up to 1000×), lowering the considerable fluorescence background that is typically present	• Requires binding to a target molecule to fluoresce • Requires specific environmental parameters to perform optimally (magnesium concentration, temperature, ect.)
*Fluorescent Protein Tags (ex. GFP)*	Protein	Fluorescent proteins can be inserted into a cell line so that as a protein is expressed it fluoresces [^270]^.	• Proteins are produced directly by the cell • 100% labeling efficiency	• These protein labels are bulky and can change protein dynamics and function.
*HaloTag and SNAP-tag*	Protein	Self-labeling protein tags such as HaloTag and SNAP-tag ^[237,238]^ are organic protein tags that can be inserted into cloning vectors [^237]^, allowing for a specific binding site for fluorophores.	• Can be used with a wide range of fluorophores • Improved brightness and photostability • Self-labeling	• Does not have 100% labeling efficiency, therefore “dark” or unlabeled proteins sometimes occur • Requires gene editing
*Fluorescent Antibody Fragments (Fabs)*	Protein	This is a technique that uses monoclonal antibodies which lack the Fc component to specifically tag proteins of interest [^271]^. The fluorophore is conjugated to a single chain antibody specific to the protein of interest [^272]^.	• Ideal method of quantifying the timing of post-translational modifications and their effects in living cells	• Challenging to design probes • Low yield when designing Fabs

The newest addition to genome editing, CRISPR, has revolutionized our ability to edit the genome as well as visualize it. Deactivated Cas9 (dCas9) provides the technology necessary to document the dynamic properties of different gene loci simultaneously [221–228]. dCas9 uses the CRISPR gene editing system for DNA labeling with a fluorescently tagged Cas9 in combination with specifically engineered guide RNAs (gRNA). This method can be used to successfully image multiple gene loci simultaneously within a living cell, which makes it an ideal labeling method for studying chromatin dynamics during mechanical stimulation [226]. One of the major challenges with CRISPR/dCas9 systems is sensitivity of detection. Most of the approaches are only successful for repetitive DNA sequences in which a single gRNA can result in labeling with numerous GFP-dCas9 proteins. Similarly, dCas13, a molecule like dCas9, targets complementary sequences of RNA. Together, the gRNA and dCas13 protein can locate a specific sequence of RNA and fluorescently label it. While this method of RNA labeling is still under development, it promises a versatile method for labeling RNAs which have not been modified through the insertion of an RNA hairpin or other sequence. In this system, either the gRNA [222] or dCas13 molecule [229] may be fluorescently labeled. Like dCas9, it suffers from low affinity but that can be overcome through multimerization of the guide RNAs. Now, specific sequences of RNA can be labeled for real-time imaging and tracking [229].

Another newer option for live-cell imaging of RNA are RNA aptamers like RNA Mango [230], RNA Spinach [231], and RNA Broccoli [232]. RNA aptamers are sequences designed as molecular beacons and selected through SELEX [233,234]. The resulting aptamer is capable of binding specific fluorophore derivatives with nanomolar affinity. This results in an increased fluorescence of up to 1000-fold. The main advantage of this method is that it provides a fluorescence enhancement upon binding, lowering the considerable fluorescence background that is typically present in other methods such as dCas9 and dCas13. This technology for visualization of RNA Mango has been used in conjunction with single-molecule fluorescence microscopy on a wide range of projects including visualizing RNA complexes in live *C. elegans* [235] and protein tyrosine kinase activity [236]. While this method is still very new, it holds promise for visualizing RNA dynamics as no other label has, providing invaluable information of the inner workings of the nucleus and the results of mechanostimulus on the transcriptome. Additional tools that have been developed recently for advanced protein imaging studies are self-labeling protein tags such as HaloTag and SNAP-tag [237,238]. These self-labeling organic protein tags can be inserted into cloning vectors [237], allowing for a specific binding site for fluorophores. The SNAP-tag and HaloTag technology can be used with a wide range of fluorophores, allowing for more flexibility than with fluorescent proteins alone. They are often used in conjunction with small, membrane permeable chemically derived dyes like ‘Janelia Fluor’ (JF) dyes that are known to be highly photostable [239]. There are many labeling options available (Table 3), but the ones described above CRISPR/Cas, RNA Aptamers, and HaloTag promise to be the most valuable for characterizing the dynamics of DNA, RNA, and protein while the nucleus is undergoing mechanical perturbations.

## Conclusion

Recent advances in the field of nuclear mechanobiology clearly indicates that the nucleus is not a passive element but actively participates in regulating cell phenotype in response to extracellular and cytoskeletal mechanical cues. As highlighted in this review, large numbers of proteins as well as inter-related structural and signaling events propose a daunting task for researchers who like to study the mechanical basis of nuclear function. While many studies focus on simplifying assumptions, mechanistic understanding of nuclear mechanobiology requires inherently complex live-cell approaches that utilize innovative experimental designs using versatile model systems such as mesenchymal stem cells that rely on reconfigurations chromatin and nucleoskeleton for their differentiation programs. Further, some of the methods highlighted here provide a high level of control on cell geometrical constraints as well as applying precise dynamic mechanical forces. Therefore, uniquely combining powerful models with experimental mechanics such as ‘deformation microscopy’ and with state-of-the-art visualization techniques to track mRNA transcription within a gene loci should yield currently unstudied correlations between subnuclear mechanics and mRNA transcription and significantly advance the current scientific knowledge in how external mechanical force regulates cell function by altering nuclear interior.
